# P25 Evaluating compliance with MHRA fluoroquinolone counselling requirements at Addenbrooke’s Hospital

**DOI:** 10.1093/jacamr/dlag102.031

**Published:** 2026-06-26

**Authors:** H Jerram, E Antomy, C Aherne, S Burrows, D Greaves

**Affiliations:** Department of Pharmacy, Addenbrooke's Hospital, Cambridge, UK; Department of Microbiology, Addenbrooke's Hospital, Cambridge, UK; Department of Pharmacy, Addenbrooke's Hospital, Cambridge, UK; Department of Microbiology, Addenbrooke's Hospital, Cambridge, UK; Department of Pharmacy, Addenbrooke's Hospital, Cambridge, UK; Department of Microbiology, Addenbrooke's Hospital, Cambridge, UK; Department of Pharmacy, Addenbrooke's Hospital, Cambridge, UK; Department of Microbiology, Addenbrooke's Hospital, Cambridge, UK; Department of Pharmacy, Addenbrooke's Hospital, Cambridge, UK; Department of Microbiology, Addenbrooke's Hospital, Cambridge, UK

## Abstract

**Background:**

Fluoroquinolones are broad spectrum antibiotics frequently used to treat a range of infections. Since 2018, the MHRA has issued multiple safety alerts highlighting potentially disabling effects on muscles, tendons, joints and the nervous system. Updated guidance requires prescribers to counsel patients on these risks and provide the MHRA information leaflet to support informed decision making (1). Fluoroquinolones remain in use across the trust, and although counselling compliance had not been formally evaluated, it was suspected to be suboptimal posing a potential medicines safety risk. Pharmacists are well placed to monitor and encourage adherence to national guidance, thereby minimizing preventable harm.

**Objectives:**

To evaluate compliance with MHRA fluoroquinolone counselling requirements. To: (i) determine the percentage of patients prescribed a fluoroquinolone who received counselling; (ii) assess whether counselling addressed key MHRA safety concerns (i.e. tendonitis, joint pain, sensory disturbances and psychiatric adverse effects); (iii) determine the percentage of patients who received an MHRA fluoroquinolone patient information leaflet; and (iv) assess whether fluoroquinolones were prescribed only when other antibiotics were unsuitable.

**Methods:**

A retrospective review of 193 fluoroquinolone prescriptions issued across November 2024 was conducted using data from the trust’s electronic prescribing system. Data on levofloxacin, ciprofloxacin, moxifloxacin, and ofloxacin (oral, injection and inhalation) were extracted and filtered using a predefined exclusion criteria. Duplicate and repeat prescriptions within the same course were removed. Data collectors reviewed records for documented counselling, discussion of MHRA specified side effects, provision of the patient information leaflet, prescribing indication, adherence to trust guidelines, profession of documenting clinician, and timing of counselling. Predefined search terms ensured consistent data collection. Data was then collated and analysed using Excel.

**Results:**

Documented counselling was present in 10% (20/193) of fluoroquinolone prescriptions, however none (0/193) addressed all MHRA specified adverse effects. Counselling was mostly performed by the prescriber (18/20), primarily doctors (17/20) and one prescribing podiatrist (1/20), in 10% (2/20) of cases counselling was completed by non-prescribing pharmacists. Counselling occurred within 24 h of prescribing in 80% (16/20) of cases. Only 7% (13/193) of patients received the MHRA leaflet, with documentation recorded in the notes. Overall, 77% (148/193) of prescriptions adhered to trust microbiology guidelines.

**Conclusions:**

Compliance with MHRA fluoroquinolone counselling guidance was poor, with no cases demonstrating complete documentation of specified safety concerns and only 7% recording leaflet provision. This indicates counselling is not consistently embedded at the point of prescribing, where shared decision making should occur. Although counselling may take place, lack of documentation limits assurance that informed discussions occurred. As pharmacist review typically follows treatment initiation, risks are often identified retrospectively, reducing opportunities to ensure informed consent. Given the potential for disabling adverse effects and need for prescribing justification, counselling must be integrated into prescribing frameworks to strengthen patient safety and antimicrobial stewardship.
